# Conservative and Surgical Management of Unilateral and Bilateral Internuclear Ophthalmoplegia (INO)—A Retrospective Analysis

**DOI:** 10.22599/bioj.280

**Published:** 2022-11-07

**Authors:** Joshua Simmons, Martin Rhodes

**Affiliations:** 1 Royal Hallamshire Hospital, Sheffield Teaching Hospitals NHS Foundation Trust, GB

**Keywords:** Internuclear Ophthalmoplegia (INO), wall-eyed bilateral internuclear ophthalmoplegia (WEBINO), strabismus, orthoptics, surgery

## Abstract

**Aim:**

To report the outcomes of the natural progression and ophthalmic treatment of patients reviewed in a tertiary hospital trust with unilateral or bilateral internuclear ophthalmoplegia.

**Method:**

A retrospective case note analysis was performed and 33 patients diagnosed with unilateral or bilateral internuclear ophthalmoplegia (INO) were identified. The diagnosis, aetiology, presence of diplopia, ophthalmic management options and progression were recorded and analysed. This included both conservative and surgical management.

**Results:**

The most common aetiologies of INO within this cohort were stroke/ischaemic (69.7%) and multiple sclerosis (MS) (30.3%). Unilateral INO was more prevalent than bilateral INO, with 20 cases (60.6%) compared to 13 cases (39.4%), respectively. A higher proportion of unilateral INO were attributed to stroke (90%) whilst a higher proportion of bilateral INO were attributed to MS (61.5%). The most prescribed management at primary assessment was occlusion (45.5%) and prisms (24.2%). Some patients required no orthoptic intervention (30.3%). Two patients had surgical management of strabismus secondary to bilateral INO.

**Conclusion:**

Occlusion was the most common form of management for symptomatic relief of diplopia. Patients who presented at the first visit with no symptoms were unlikely to need any orthoptic intervention. Of the two patients who went on to require surgical intervention, restoration of binocular single vision (BSV) was achieved post-operatively with the use of a Fresnel prism. However, the differences in both surgical technique and number of surgeries required make this difficult to generalise. Additional research is needed to further explore the surgical management of INO.

## Introduction

Internuclear ophthalmoplegia (INO) consists of an ipsilateral adduction deficit, with abducting nystagmus of the contralateral eye, caused by a lesion in the medial longitudinal fasciculus (MLF) (Leigh & Zee, 2006). Other typical clinical features of INO include an impairment of saccadic movement, notably the adducting saccades, and impairment of optokinetic nystagmus (OKN) (Young, 1995; Ansons & Davis, 2014). Patients with INO typically present with exophoria (X) or exotropia (XT), which increases when attempting to look in the direction of action of the affected medial rectus and results in symptoms of horizontal diplopia and oscillopsia (Nathan & Donahue, 2012). Patients with INO may also have abnormal vertical eye movements, including vertical nystagmus and skew deviation (Leigh & Zee, 2006). Bilateral INO with XT in primary position is commonly known as wall-eyed bilateral internuclear ophthalmoplegia (WEBINO). In adults, the most common aetiology of INO is infarction, ischaemia and multiple sclerosis (MS), although head trauma, neoplasms, inflammatory or viral factors have also been reported (Keane, 2005). Unilateral INO is more frequently related to ischaemic events, whereas bilateral INO is more commonly associated with MS (Wu, Cafiero-Chin & Marques, 2015).

As spontaneous improvement is common in INO (Nathan & Donahue, 2012), management in the early stages is typically conservative. This includes management of symptomatic diplopia, with prisms or occlusion. Several studies have looked at the effect of other treatment for INO, including surgery and botulinum toxin (BT).

Roper-Hall, Cruz & Chung (2008) retrospectively reviewed a series of eight patients with bilateral INO and large XT, range 25 – >100 prism dioptres (PD), who underwent extraocular muscle surgery. Postoperatively, there was an 85% reduction in the exo-deviation, with seven of the eight patients regaining some level of BSV with or without prism.

Adams, Leavitt & Holmes (2009) reported three patients with WEBINO due to MS who underwent surgery for large angle XT and symptomatic diplopia. Post-operatively, all three patients experienced resolution of the diplopia. However, one patient required a second surgery for recurrence of XT and bilateral INO.

Murthy et al. (2007) observed a series of 16 patients who underwent BT injections for management of diplopia and eye alignment. Whilst BT improved diplopia symptoms and eye alignment, only three patients were discharged following BT, while five continued BT injections, five received occlusion, one required a prism and two required strabismus surgery.

This study retrospectively reviewed a cohort of patients with unilateral and bilateral INO to evaluate the natural progression and ophthalmic treatment received. The outcome of surgical intervention in two patients with poor response to conservative treatment is presented.

## Method

Local trust approval was granted as the study was a retrospective case note analysis. Ethical approval was not required. The records of 33 patients at a tertiary hospital trust, diagnosed with INO (unilateral or bilateral) between April 2008 and August 2016, were reviewed to identify the progression and management of INO, using both the orthoptic and ophthalmology reports. Diagnosis of INO/WEBINO was made following first orthoptic assessment. Examination included case history, visual acuity, cover test, measurement of the deviation, assessment of binocular single vision and assessment of ocular motility. Information gathered included the age and sex of the patient, cause of the INO, type of deviation and angle of deviation in primary position, degree of restriction of ocular motility and treatment/management given.

## Results

### Clinical demographics and aetiology

The results of the cohort (n = 33) are displayed in Table 1. Seventeen patients (51.5%) were male and 16 (48.5%) were female. The average age at presentation was 59.9 years. Unilateral INO was more prevalent than bilateral INO, with 20 cases (60.6%) in comparison to 13 cases (39.4%), respectively. When examining the laterality of unilateral INO, right INO (75%) was more commonly found than left INO (25%).

**Table 1:** Summary of the findings of case note review, including diagnosis, outcomes and management of patients diagnosed with INO and WEBINO.


		NUMBER OF PATIENTS (PERCENTAGES)

Total Cohort Number	33	

Outcomes at First Assessment	Occlusion	15 (45.5%)

	Prism	8 (24.2%)

	Nil	10 (30.3%)

Aetiology	Stroke	23 (69.7%)

	Multiple Sclerosis	10 (30.3%)

Diagnosis	INO (Unilateral)	20 (60.6%)

	WEBINO (Bilateral)	13 (39.4%)

Laterality (in INO)	Right	15 (75%)

	Left	5 (25%)

Reported Diplopia?	Yes	21 (63.6%)

	No	12 (36.4%)

INO Management	Occlusion	10 (50%)

	Prism	4 (20%)

	Nil	6 (30%)

WEBINO Management	Occlusion	5 (38.4%)

	Prism	4 (30.8%)

	Nil	4 (30.8%)


In the overall cohort (n = 33) the most common aetiologies of INO were stroke/infarction (n = 23, 69.7%) and MS (n = 10, 30.3%). In unilateral INO (n = 20), most cases were attributed to stroke (90.0%), with MS only accounting for 10% of cases. Comparatively, most cases of bilateral INO were due to MS (61.5%) and fewer were due to stroke (38.5%). Of those with INO secondary to MS, the average age was 45.7 years, and in stroke/infarction, the average age was 66.0 years.

### Management of INO

**Figures 1–2:**
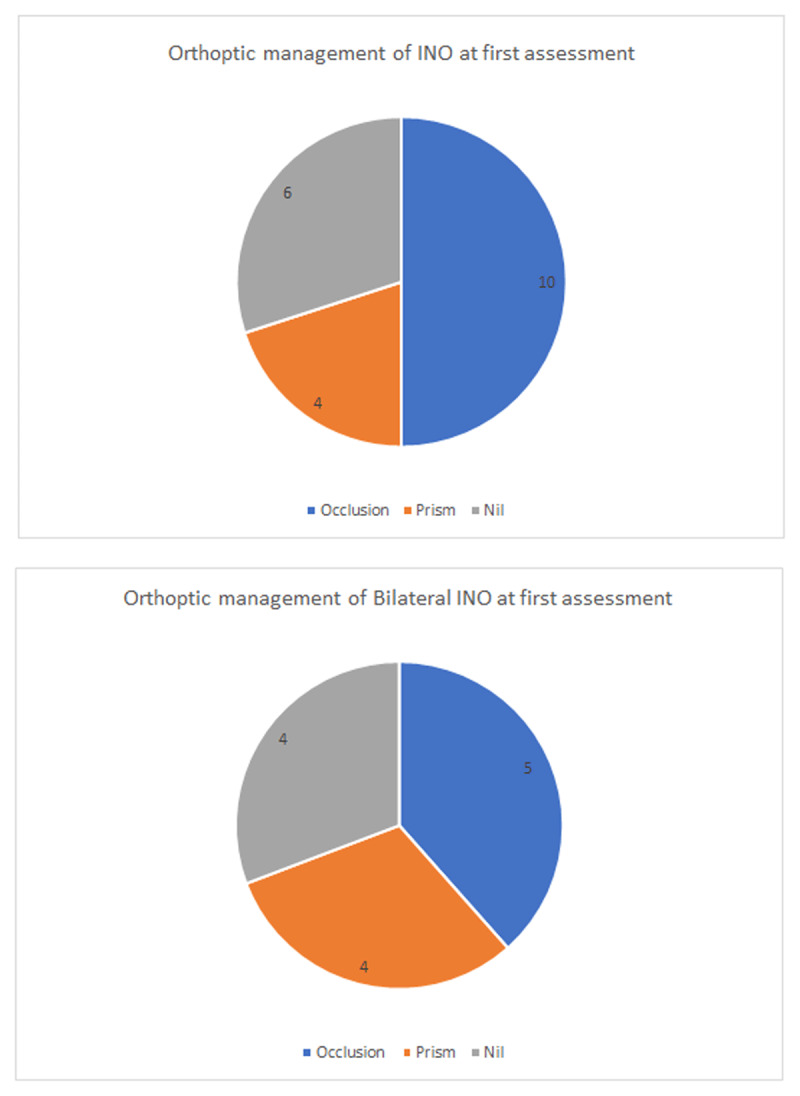
Orthoptic management, showing number of patients, of internuclear ophthalmoplegia and bilateral internuclear ophthalmoplegia at first assessment.

The most common method of Orthoptic management for INO at visit one was occlusion. Ten unilateral INO cases (50%) and five bilateral INO cases (38.4%) received occlusion at the first visit (Figures 1–2). A larger proportion (50%) of patients diagnosed with unilateral INO required occlusion for symptomatic diplopia, in contrast to patients diagnosed with bilateral INO (38.4%.) Ten patients required no orthoptic intervention at the first assessment (30% of INO patients and 30.8% of WEBINO patients).

### Orthoptic findings

Most patients demonstrated an XT for both near and distance fixation (n = 19 and 18, respectively) (Table 2). However, some patients demonstrated other manifest or latent strabismus, with some showing no deviation in primary position. In those with an XT in primary position, the mean angles of deviation were 20.5 PD (near) and 15.4 PD (distance) in unilateral INO and 55.6 PD (near) and 62.0 PD (distance) in bilateral INO/WEBINO. An associated skew deviation was diagnosed in six cases (18.2%); four of these (20%) had unilateral INO and two (15.4%) had bilateral INO. Of the six cases identified with skew deviation, five (83.3%) had an aetiology of stroke. Abducting nystagmus was documented in 27 (81.8%) patients; however, this was not specifically documented (or negatively recorded) in all patients, meaning that it was unclear if the patient did not have abducting nystagmus in all other cases. Fifteen patients (75.0%) with unilateral INO and 12 patients (92.3%) with bilateral INO had documented abducting nystagmus at their first assessment. Of these cases, 37% (n = 10) had an aetiology of MS and 63% (n = 17) had an aetiology of stroke.

**Table 2:** Summary of the findings of the cover test results and angle of deviation measurements for near and distance fixation in our cohort of patients diagnosed with INO/WEBINO. X: Exophoria, XT: Exotropia, E: Esophoria, ET: Esotropia, NAD: No apparent deviation, PD: Prism dioptres.


COVER TEST FINDINGS FOR NEAR FIXATION	TYPE OF STRABISMUS	NUMBER OF PATIENTS (PERCENTAGE)	MEAN ANGLE OF DEVIATION IN PRISM DIOPTRES (PD) (AVERAGE)	RANGE OF ANGLE OF DEVIATION MEASUREMENT IN PRISM DIOPTRES (PD)

	X	7 (21.2%)	47.4 PD	2–12

	XT	19 (57.6%)	18 PD	4–100

	E	0 (0%)	/	/

	ET	6 (18.2%)	14.8 PD	5–20

	NAD	1 (3.0%)	0	0

**COVER TEST FINDINGS FOR DISTANCE FIXATION**				

	X	6 (18.2%)	46.8 PD	1–5

	XT	18 (54.5%)	23.4 PD	4–100

	E	0 (0%)	/	/

	ET	8 (24.2%)	5.3 PD	4–20

	NAD	1 (3.0%)	0	0


Due to the retrospective nature of the study, some information was not available or not recorded at every visit. A large proportion of patients were seen as inpatients on the stroke wards, which limited the assessment possible. For example, prism cover test (PCT) measurement of the angle of deviation in different positions of gaze was not always completed, or the method of assessment of BSV varied.

### Follow up

Of the 10 patients who did not require any management at visit one, five patients did not attend for any follow-up, two patients self-discharged, two patients were discharged from the orthoptic clinic (after two visits), and one patient went on to develop symptomatic diplopia due to decompensating esophoria.

Due to the limitations of conservative management of symptomatic diplopia in large angle XT, two patients had later surgical correction of XT secondary to WEBINO, the results of which are discussed below.

### Surgical Case One

This patient had bilateral INO due to cerebellar infarct. Initial assessment was as an inpatient on the stroke ward. They had no known previous ophthalmic history. A cover test revealed large angle XT (approximately 80 PD) with abducting nystagmus on both dextro and laevo-versions. The patient reported symptomatic diplopia and oscillopsia, which were treated with occlusion. Follow-up was initially as an inpatient, and then as an outpatient before being referred into the strabismus service for consideration of surgical correction of the XT. At approximately 18 months post stroke, the angle of deviation had stabilised, and symptomatic diplopia was being managed by a Fresnel prism (20 PD base in over the left eye (BI LE) and 3 PD base down over the right eye (BD RE)). Without prismatic correction, the patient demonstrated a left XT with left hypotropia (LHoT). Ocular motility assessment demonstrated bilateral medial rectus (MR) restrictions (–0.5 right MR, –2.5 left MR) with bilateral abducting nystagmus. No compensatory head posture was present. The patient was listed for strabismus surgery.

Pre-operatively (23 months post-stroke), the patient had 35 PD XT (near) and 20 PD XT and 3 PD LHoT (distance). Bilateral MR restriction were graded as –0.5 right MR and –2 left MR. Right lateral rectus (LR) recession to 13.5 mm from the limbus, and the left MR resection of 3.5 mm was performed.

One day post-operatively, the patient had a small angle right hypertropia (RHT) for near (5 PD RHT) with a small right esotropia with hypertropia for distance fixation (5 PD ET and 4 PD RHT). MR limitations were unchanged (–0.5 right MR, –2 left MR). A small vertical Fresnel prism (3 BD RE near, 4 BD RE distance) was used to join symptomatic diplopia and restore BSV. Two weeks post-operatively, the patient had a minimal right ET near and distance (4 PD ET) and was fitted with a Fresnel prism.

The patient continued to attend the Orthoptic department for assessment of ocular motility and treatment of diplopia with a small Fresnel prism. Ten months post-operatively, the patient had experienced a six-month period of stability and prisms were incorporated into their glasses. With incorporated prisms the patient had proven BSV (sensory fusion and stereopsis), was asymptomatic with no diplopia and was discharged from the Orthoptic clinic.

### Surgical Case Two

This patient had bilateral INO due to cerebellar and occipital infarcts. Initial assessment was as an inpatient on the stroke ward. They had no known previous ophthalmic history. The patient had a large angle right XT and symptomatic diplopia, which was treated with occlusion. Stabilisation of the angle of deviation took 18 months.

At pre-operative assessment, the patient had 55–60 PD XT with slight RHT (near) and 55 PD XT with slight RHT (distance). The vertical angle of deviation was not measured pre-operatively. Bilateral MR restrictions were graded as –3.5 right and –3 right left, and abducting nystagmus was present on both dextro and laevo-versions. No compensatory head posture was used. Bilateral MR resections (6 mm) and bilateral LR recessions (left 4.5 mm and right 5 mm) were performed with infra-placement of both MR insertions by 2 mm.

One day post-operatively, the patient had a slight right ET and right hypotropia (RHoT) for both near (25 PD ET and 5 PD RHoT) and distance (25 PD ET and 8 PD RHoT.) The patient was fitted with a Fresnel prism, which allowed the patient to regain BSV (Bagolini glasses). The adduction deficits had been decreased to –0.5 in the right and –1 in the left.

Over the course of the following 6 months, the patient continued to attend for Orthoptic follow up, still showing symptomatic right ET and RHoT. The patient was listed for further surgery.

Pre-operatively, the angle of deviation was 14 PD ET and 5 PD RHoT (near) and 16 PD ET and 5 PD RHoT (distance) fixation, with adduction restrictions of –1.0 right and left. The patient underwent a right LR advancement (5 mm) with vertical transposition upward of the right LR and MR (2 mm).

One day post-operatively, the patient had a residual RHoT at near (5 PD RHoT) and distance (3 PD RHoT). They were fitted with a Fresnel prism to join their diplopia and restore BSV. Over the following few months, the patient continued to show stability and was happy with the prism they were given.

## Discussion

The majority of our cohort of patients diagnosed with unilateral or bilateral INO required occlusion treatment to help with symptomatic diplopia on visit one. A large number of these patients (n = 20, 60.6%) were seen on the ward as inpatients following stroke, and so ocular assessment took place very soon after the onset of symptoms. This may account for the generally large number of patients requiring occlusion after their first examination. This early conservative treatment option in INO was in agreement with the findings of Nathan & Donahue (2012). In our cohort, approximately one third did not require orthoptic intervention at their first visit (INO 30.0%, WEBINO 30.8%).

Only one patient who required no treatment on the first visit required any form of treatment at follow-up visits. In this case, the patient in question required a prism to maintain BSV, and this was not attributed to the INO, but to a decompensating esophoria. All other patients found to need no management on visit one, remained this way, and needed no orthoptic management. This suggested that if patients are asymptomatic at the initial visit, they are not likely to develop any further symptoms or require any further orthoptic management. This was similar to the results of Eggenberger et al. (2002), who concluded that most patients diagnosed with INO with an ischaemic aetiology became asymptomatic in primary position over two to three months.

In our cohort, six patients were also found to have an associated skew deviation. Four of these patients had unilateral INO. This supports other reports of skew deviation commonly occurring with unilateral INO (Leigh & Zee, 2006). Although a large number within the sample demonstrated an XT on cover testing, not all patients were found to have XT. This is in agreement with the findings of Johkura et al. (2015), who reported that XT, although common in the acute stages of INO, was not present in their entire cohort, and that a number of those initially presenting with XT upon admission showed resolution of XT within one week. Due to the variability between referral to the Orthoptic assessment, it may be that some of the patients had improved since their initial presentation.

A study by Durnian et al. (2009) reported that the most common procedure performed for surgical management of INO was a bilateral LR recession, followed by a unilateral LR recession. Furthermore, a small number underwent a unilateral LR recession with MR resection. Nelson & Peragallo (2022) retrospectively analysed the surgical results of seven patients who had surgery for XT with INO, using a range of surgical techniques, including unilateral recess with plication and recess/resect, among others. They commented that the variable approaches taken to surgical methods, combined with various pre-operative factors, made it difficult to analyse outcomes. Although only two patients in our cohort of 33 underwent strabismus surgery, the surgical procedures performed were in line with the procedures recommended by others (Durnian, et al 2009; Nelson and Peragallo, 2022).

In our study, case one underwent one surgical procedure and case two underwent two surgical procedures. Both cases had a good outcome post-operatively, achieving BSV with a small amount of Fresnel prism, which was later incorporated into their glasses. This demonstrates an improved binocular outcome following surgical intervention. Whilst both cases had bilateral INO secondary to stroke, the results are difficult to generalise to all cases of INO due to the limited sample size, and the variability between cases. Further study of surgical outcomes in unilateral INO and INO secondary to MS would be interesting to study in the future.

It is acknowledged that there are limitations in a retrospective case series such as this. The small sample size makes it hard to generalise data amongst a larger INO population. Some data were missing due to tests that were not performed or results that were not recorded, particularly the absence of clinical findings such as abducting nystagmus. Missing data is a potential confounding variable. A number of patients were first seen as inpatients on the stroke ward, which limited the orthoptic assessment and standardisation of some testing. In our tertiary referral centre, most inpatients are referred to the orthoptic team very soon after the onset of symptoms and diagnosis of stroke. This stroke cohort typically have multiple health conditions which can impact their ability to carry out a complete and accurate assessment at the first visit. This, however, should be considered in the context of orthoptists as autonomous practitioners, expertly performing a tailored assessment for each individual cases and providing therapy or advice for patients who are suffering from symptomatic diplopia secondary to INO/WEBINO at the earliest opportunity. In an outpatient setting and a controlled environment, examinations and assessments are able to be more standardised. Follow up time was variable in our cohort, and a number of patients did not have any documented follow-up on record. This could be attributed to the inpatient setting and multiple health issues, which could affect attendance as an outpatient. This limited our ability to draw conclusions regarding the evolution/progression of INO and long-term outcomes, with and without treatment.

In the future, a larger prospective study investigating the conservative and surgical management of INO/WEBINO would enhance the available evidence on the management of INO, despite the challenge of the relative rarity of both the condition and the numbers that require surgical intervention. Further prospective research would be enhanced by having a consistent, standardised and more complete first assessment and follow up, where possible and practical. It is not yet known whether the surgical outcome of INO is affected by the condition being unilateral/bilateral and aetiology.

## Conclusion

In our cohort of 33 patients with INO, the most common management for symptomatic diplopia was uniocular occlusion. Patients who were asymptomatic at the first visit were unlikely to need any later Orthoptic intervention. Two of our 33 patients required later surgical intervention. In both cases, restoration of BSV was achieved post-operatively with the use of prisms, highlighting that good functional outcomes can be achieved even in those where conservative management is challenging due to the size of the XT. Due to the small numbers with INO undergoing surgery, our ability to make conclusions relating to surgery are limited. Additional research is needed to further explore the surgical management of INO/WEBINO. Further research may want to focus on a more standardised and consistent first assessment setting.
